# β-adrenergic receptor agonist promotes ductular expansion during 3,5-diethoxycarbonyl-1,4-dihydrocollidine-induced chronic liver injury

**DOI:** 10.1038/s41598-023-33882-w

**Published:** 2023-05-01

**Authors:** Naoki Tanimizu, Norihisa Ichinohe, Toshihiro Mitaka

**Affiliations:** 1 Department of Tissue Development and Regeneration, Research Institute for Frontier Medicine, Sapporo Medical University School of Medicine, S-1, W-17, Chuo-ku, Sapporo, 060-8556 Japan; 2 Present Address: Division of Regenerative Medicine, Center for Stem Cell Biology and Regenerative Medicine, The Institute of Medical Science, The University of Tokyo, 4-6-1 Shirokanedai, Minato-ku, Tokyo, 108-0071 Japan

**Keywords:** Cell biology, Hepatology

## Abstract

Intrahepatic nerves are involved in the regulation of metabolic reactions and hepatocyte-based regeneration after surgical resection, although their contribution to chronic liver injury remains unknown. Given that intrahepatic nerves are abundant in the periportal tissue, they may be correlated also with cholangiocyte-based regeneration. Here we demonstrate that isoproterenol (ISO), a β-adrenergic receptor agonist, promoted ductular expansion induced by 3,5-diethoxycarbonyl-1,4-dihydrocollidine (DDC) in vivo. Immunofluorescence analysis shows that nerve fibers positive for tyrosine hydroxylase form synaptophysin-positive nerve endings on epithelial cell adhesion molecule-positive (EpCAM^+^) cholangiocytes as well as on Thy1^+^ periportal mesenchymal cells (PMCs) that surround bile ducts, suggesting that the intrahepatic biliary tissue are targeted by sympathetic nerves. In vitro analyses indicate that ISO directly increases cAMP levels in cholangiocytes and PMCs. Mechanistically, ISO expands the lumen of cholangiocyte organoids, resulting in promotion of cholangiocyte proliferation, whereas it increases expression of *fibroblast growth factor 7*, a growth factor for cholangiocytes, in PMCs. Taken together, the results indicate that intrahepatic sympathetic nerves regulate remodeling of bile ducts during DDC-injury by the activation of β-adrenergic receptors on cholangiocytes and PMCs.

## Introduction

Peripheral nerves establish close communications with neighboring tissues and have crucial roles in tissue morphogenesis^1^. During vascular development in the skin, sensory nerves supply CXCL12 and vascular endothelial growth factor (VEGF) to induce the migration of endothelial cells and arterial differentiation, respectively^2,3^. In the submandibular gland, parasympathetic nerves induce lumen continuity of epithelial tubules by secreting vasoactive intestinal peptide^4^.

Mammalian liver tissue is innervated by autonomic nerves^5^. Although the extension of the autonomic nerve network of the liver is species dependent, nerve fibers are abundant in periportal tissue, consisting of portal vein (PV), intrahepatic bile duct (IHBD), hepatic artery, and lymphatic vessel^6–8^. We previously demonstrated that cholangiocytes, which are epithelial cells that form IHBDs, regulate the innervation and re-innervation of liver tissue during development and regeneration, respectively, by secreting nerve growth factor (NGF)^9^. However, whether intrahepatic nerves regulate morphogenesis and regeneration of bile ducts is unknown. As the biliary tubular network is established before intrahepatic innervation^9^, nerve-epithelial interactions may contribute to IHBD remodeling during chronic injury and not development.

The three-dimensional (3D) architecture of liver epithelial tissue, which consists of bile canaliculi of hepatocytes and IHBDs, is dynamically rearranged upon acute and chronic injury and during regeneration. In particular, the expansion and remodeling of IHBDs, known as ductular reactions (DRs), are frequently observed in liver tissue under conditions of chronic liver injury. Although DRs are correlated with the activation of hepatic progenitor cells^10–12^, their major physiological role is an adaptive response to cholestasis and bile duct damage^13–15^. Evidences implicating the NOTCH^16^, Hippo/YAP^17^, fibroblast growth factor 7 (FGF7)^18^, TWEAK^19^, and Wnt/β-catenin^20^ signaling pathways in DRs highlight the interactions between cholangiocytes and portal mesenchymal cells (PMCs) or macrophages. However, it remains to be clarified whether intrahepatic nerves are involved in DRs and, if so, how they regulate bile duct remodeling.

Here, we demonstrate that intrahepatic sympathetic nerves are involved in the remodeling of IHBDs in mice. Administration of the β-adrenergic receptor agonist isoproterenol (ISO) promoted DRs induced by a 3,5-diethoxycarbonyl-1,4-dihydrocollidine (DDC)-containing diet. Given that tyrosine hydroxylase^+^ (TH^+^) synaptophysin^+^ sympathetic nerve endings are observed on cholangiocytes as well as on PMCs, sympathetic nerves affect those cells in DRs. Consistently, in vitro, ISO directly acted on cholangiocytes and PMCs, which were correlated with cholangiocyte proliferation. Our results suggest that sympathetic nerves activation promotes ductular expansion in chronically injured liver through two cellular components of IHBDs.

## Results

### Characterization of intrahepatic sympathetic nerves in mice

**Figure 1:**
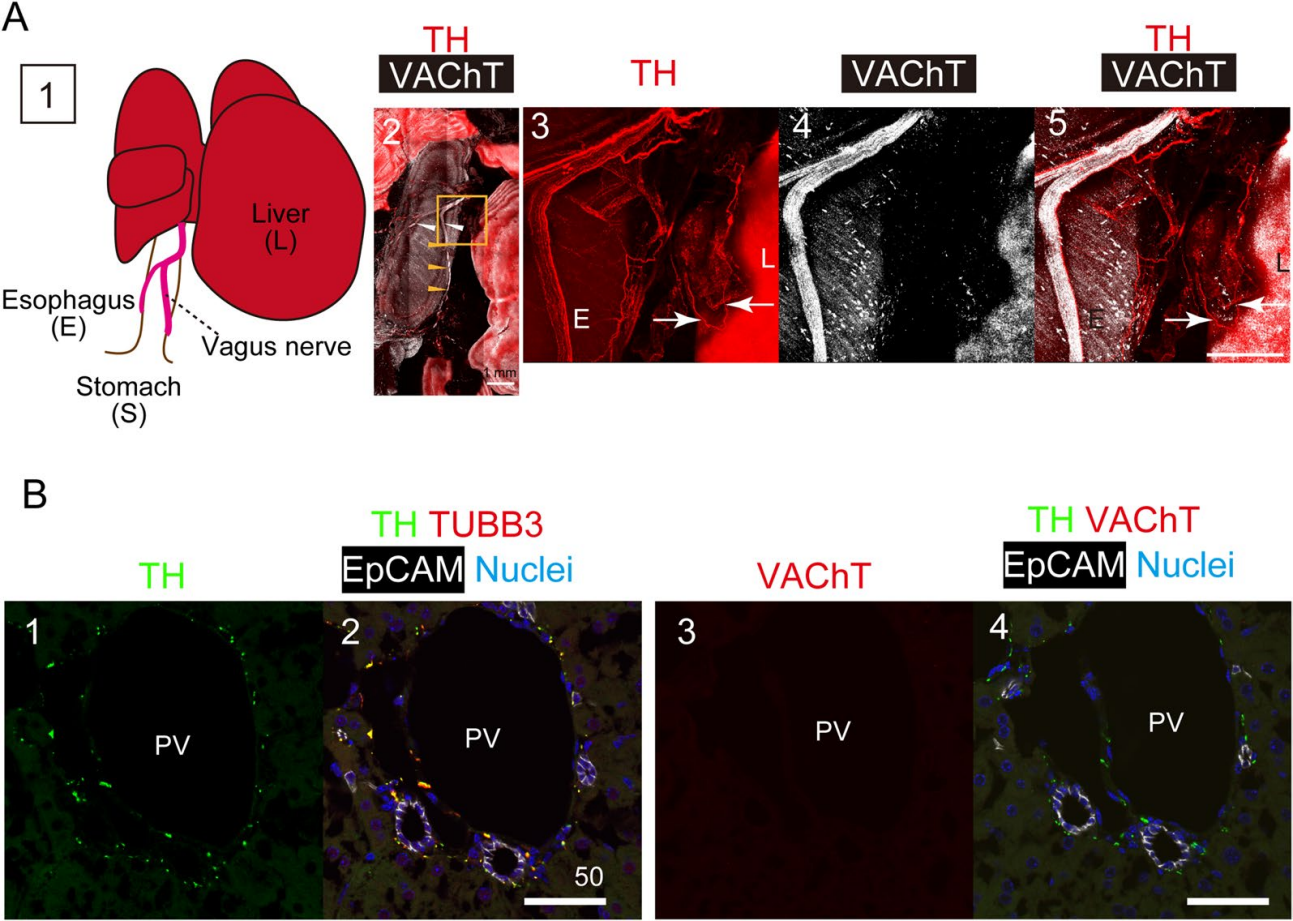
Intrahepatic sympathetic nerves form synaptic endings on cholangiocytes. (**A**) Sympathetic and parasympathetic characteristics of nerve fibers along the esophagus. Nerve fibers on the esophagus and the liver (panel 1) are characterized by immunostaining. Vagus nerves positive for vesicular acetylcholine transporter (VAChT) run along the esophagus, and sympathetic nerves positive for tyrosine hydroxylase (TH) are parallel to the vagus nerve bundle (arrowheads in panel 2). TH^+^ fibers extend toward the liver (arrows in panels 3 and 5), whereas VAchT^+^ fibers are absent from the interstitial space between the esophagus and the liver (panels 4 and 5). Esophagus samples with liver and stomach tissue isolated from adult mouse were stained with anti-VAChT (white) and anti-TH (red) antibodies. The box in panel 2 is enlarged in panels 3–5. Scale bars represent 500 μm. (**B**) Intrahepatic nerves show sympathetic characteristics. Intrahepatic nerves are positive for TUBB3^+^/TH^+^ (panels 1, 2, and 4) but negative for VAChT (panel 3). Serial sections of adult liver were stained with anti-TH (green), anti-TUBB3 (red), and anti-EpCAM (white) antibodies (panels 1 and 2), and with anti-TH, anti-VAChT, and anti-EpCAM antibodies (panels 3 and 4). Scale bars represent 100 μm. PV, portal vein.

**Figure 2:**
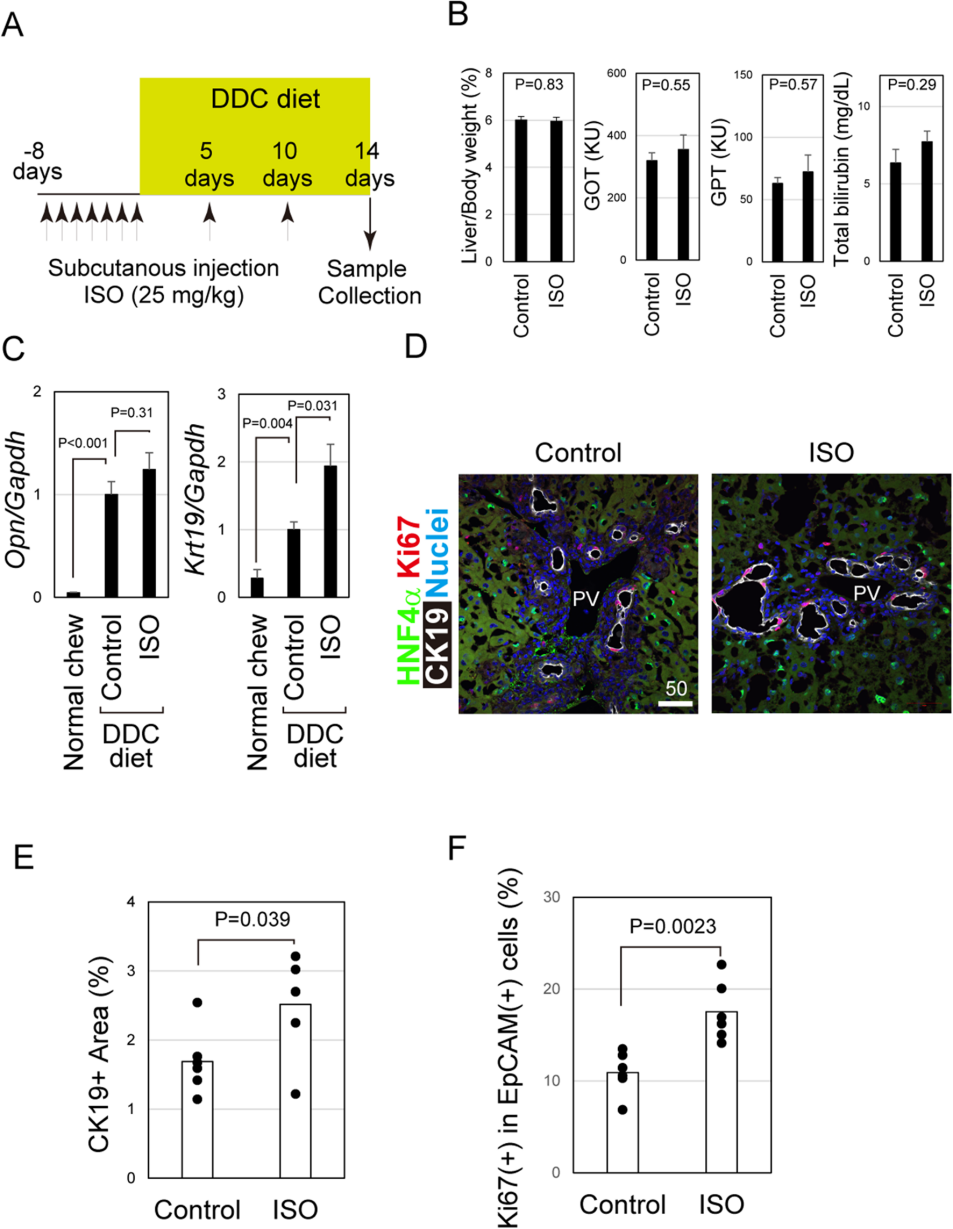
Activation of beta-adrenergic receptors (β-ADRs) promotes ductular reactions (DRs) induced by 3,5-diethoxycarbonyl-1,4-dihydrocollidine (DDC) diet. (**A**) Timing of chronic liver injury and isoproterenol (ISO) administration. ISO was subcutaneously injected daily for a week before DDC-feeding. During DDC-feeding, it was administered to mice every 5 days. Mice administered PBS (n = 6) or ISO (n = 6) were fed DDC-diet. (**B**) ISO does not affect hepatic injury induced by DDC diet. Liver/body weight as well as serum levels of GOT, GPT, and total bilirubin are not affected by ISO administration. Error bars represent SEM. (**C**) ISO increases the expression of bile duct cell markers induced by DDC diet. Quantitative PCR (qPCR) analysis demonstrates that DDC-diet increases the expression of *Opn* and *Krt19* in liver tissue, and ISO treatment further increases the expression of *Krt19*. Error bars represent SEM. (**D**) ISO enhances DRs induced by DDC diet. CK19^+^ duct structures around portal veins (PVs) are increased by ISO-administration. In both control and ISO-administered mice, Ki67^+^ cells are frequently observed in CK19^+^ cholangiocytes and infrequently in HNF4α^+^ hepatocytes. Liver sections of DDC-fed mice with or without ISO-administration were stained with anti-HNF4α, anti-CK19, and anti-Ki67 antibodies and Hoechst 33,342. Scale bars represent 50 μm. (**E**) Quantification of CK19^+^ duct expansion. CK19^+^ areas were identified and quantified in eight different fields of a liver section of each mouse. The ratio of the CK19^+^ area per field was determined for each mouse and plotted in the group. The bars represent the average values of control and ISO-administered mice. (**F**) ISO enhances cholangiocyte proliferation. Liver sections were stained with anti-CK19 and anti-Ki67 antibodies and Hoechst 33,342. The number of CK19^+^ cells negative and positive for Ki67^+^ were counted. The ratio of Ki67^+^/CK19^+^ cells to all counted CK19^+^ cells was determined for each mouse and plotted in the group. The bars represent the average values of control and ISO-administered mice.

**Figure 3:**
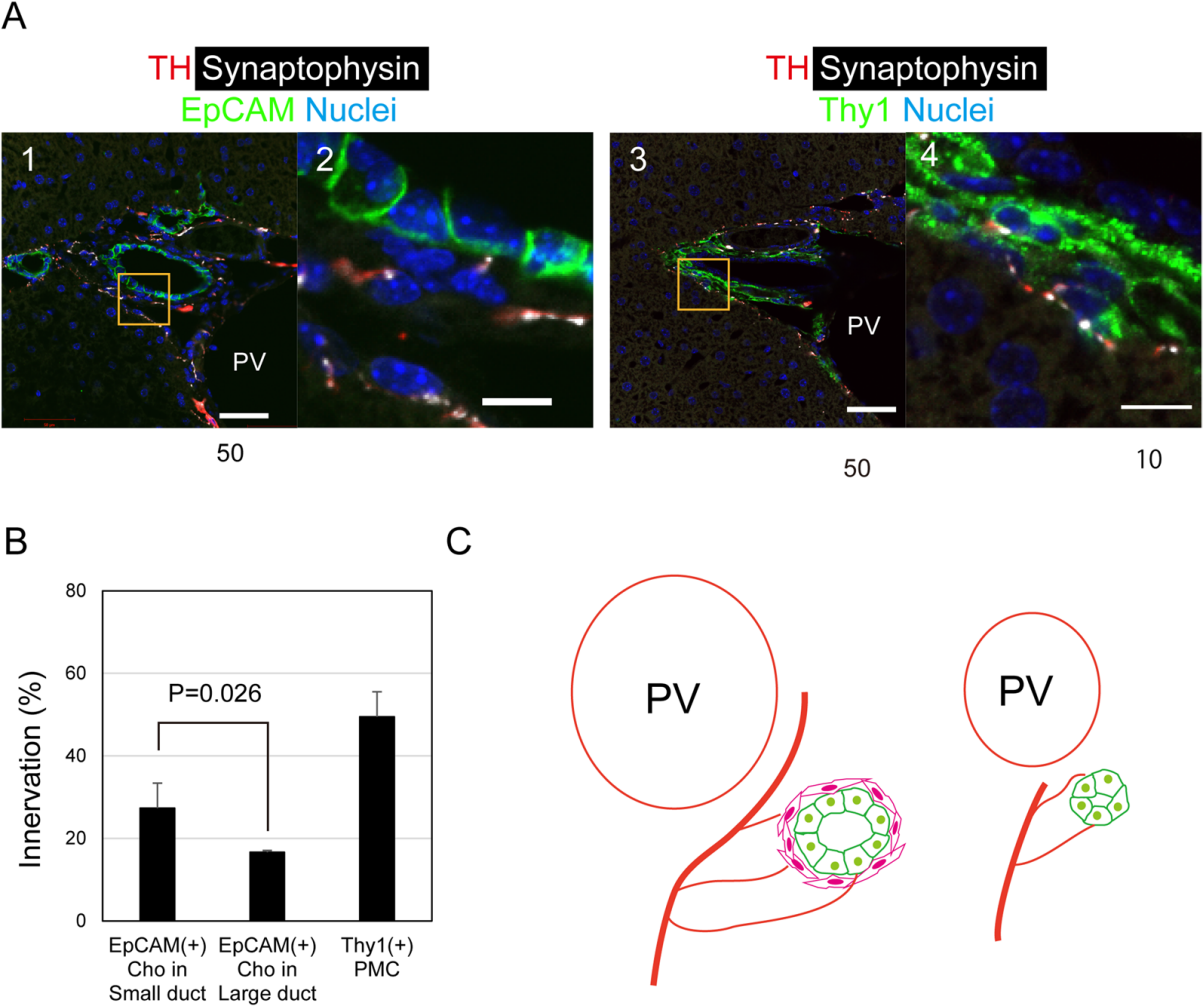
Sympathetic nerves form nerve endings on cholangiocytes and periportal mesenchymal cells. (**A**) Cholangiocytes and portal mesenchymal cells (PMCs) are innervated by TH^+^ nerves. The terminal of a TH^+^ nerve fiber (green) positive for synaptophysin (SYN) (white) is located just beneath the basal domain of EpCAM^+^ cholangiocytes (panels 1 and 2) as well as near Thy1^+^ PMCs. Boxes in panels 1 and 3 are enlarged in panels 2 and 4. Bars represent 50 and 10 μm in panels 1 and 3, and panels 2 and 4, respectively. (**B**) Proportion of cholangiocytes and PMCs associated with SYN + nerve endings. Small bile ducts are more directly associated with SYN^+^ nerve endings as compared with large ones. Large bile ducts are surrounded by Thy1^+^ PMCs, which are mostly associated with SYN^+^ nerve endings. (**C**) Schematic view of the interaction between sympathetic nerves and bile ducts. Large bile ducts surrounded by PMCs are located near the liver hilum, where sympathetic nerves dominantly make their ending near PMCs rather than cholangiocytes. On the other hand, sympathetic nerves directly interact with cholangiocytes in small ductules, which are located in the peripheral liver tissue, more frequently as compared with those in large ducts.

**Figure 4:**
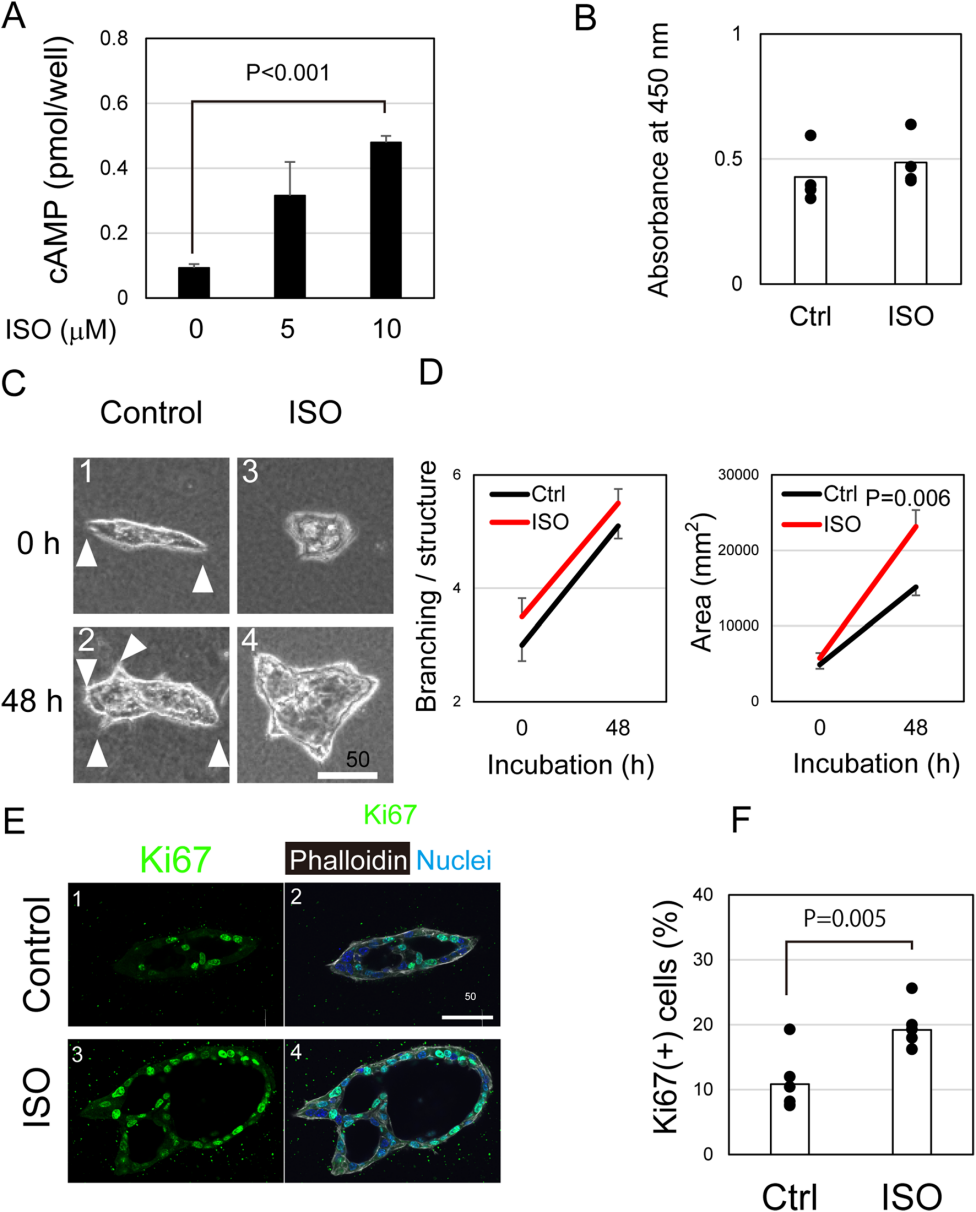
Activation of beta-adrenergic receptors (β-ADRs) expands the luminal structure of cholangiocyte organoids and promotes cholangiocyte proliferation. (**A**) Isoproterenol (ISO) increases intracellular cAMP in cholangiocytes. Intracellular cAMP levels are significantly increased in the presence of 10 μM ISO. The average values of three wells (n = 3) of each condition are shown in the graph. Error bars represent SEM. (**B**) ISO does not affect cholangiocyte proliferation. EpCAM^+^ cholangiocytes were cultured for a week with or without 10 μM ISO. Cells were further incubated in the presence of WST-1 for 60 min, and then the absorbance at 450 nm was measured. The average value of three wells (n = 3) of each condition are shown in the graph. Bars represent SEM. (**C**) ISO promotes the formation of cholangiocyte organoids. Cholangiocytes form organoids with branching structures (arrowheads in panels 1 and 2). ISO promotes the expansion of organoids (panels 3 and 4). A week after 3D culture in type I collagen gel, the medium was replaced with that containing 10 μM ISO. The scale bar represents 50 μm. (**D**) Effect of ISO on cholangiocyte organoid structure. ISO has no effect on cholangiocyte organoid branching (left panel), whereas it promotes their expansion (right panel). Cholangiocyte organoids expand in the presence of EGF and HGF, and ISO further promotes this expansion. After 7 days of cell culture, ISO was added to the culture and incubated for an additional 2 days. The average values of organoid size (n = 12–15) in each condition are shown in this graph. Cultures were repeated four times and representative data are graphically shown. (**E**) ISO promotes lumen expansion of cholangiocyte organoids. Cholangiocyte organoids are associated with luminal structures (panels 1 and 2), and ISO expands the lumen (panels 3 and 4). In both conditions, organoids contain Ki67^+^ proliferating cells. Organoids were stained with an anti-Ki67 antibody, phalloidin, and Hoechst 33,342. The scale bar represents 50 μm. (**F**) ISO increases cholangiocyte proliferation. ISO increases the ratio of Ki67^+^ proliferating cells. Organoids were stained with an anti-Ki67 antibody and Hoechst 33,342. The number of nuclei positive and negative for Ki67 was counted and the ratio of Ki67^+^ cells to all counted cells are shown in the graph. Three to five organoids in each culture well were used for Ki67 staining. Each culture was independently repeated three times. A total of 11 organoids was used to count Ki67^+^ cells (n = 11). Bars represent SEM.

**Figure 5:**
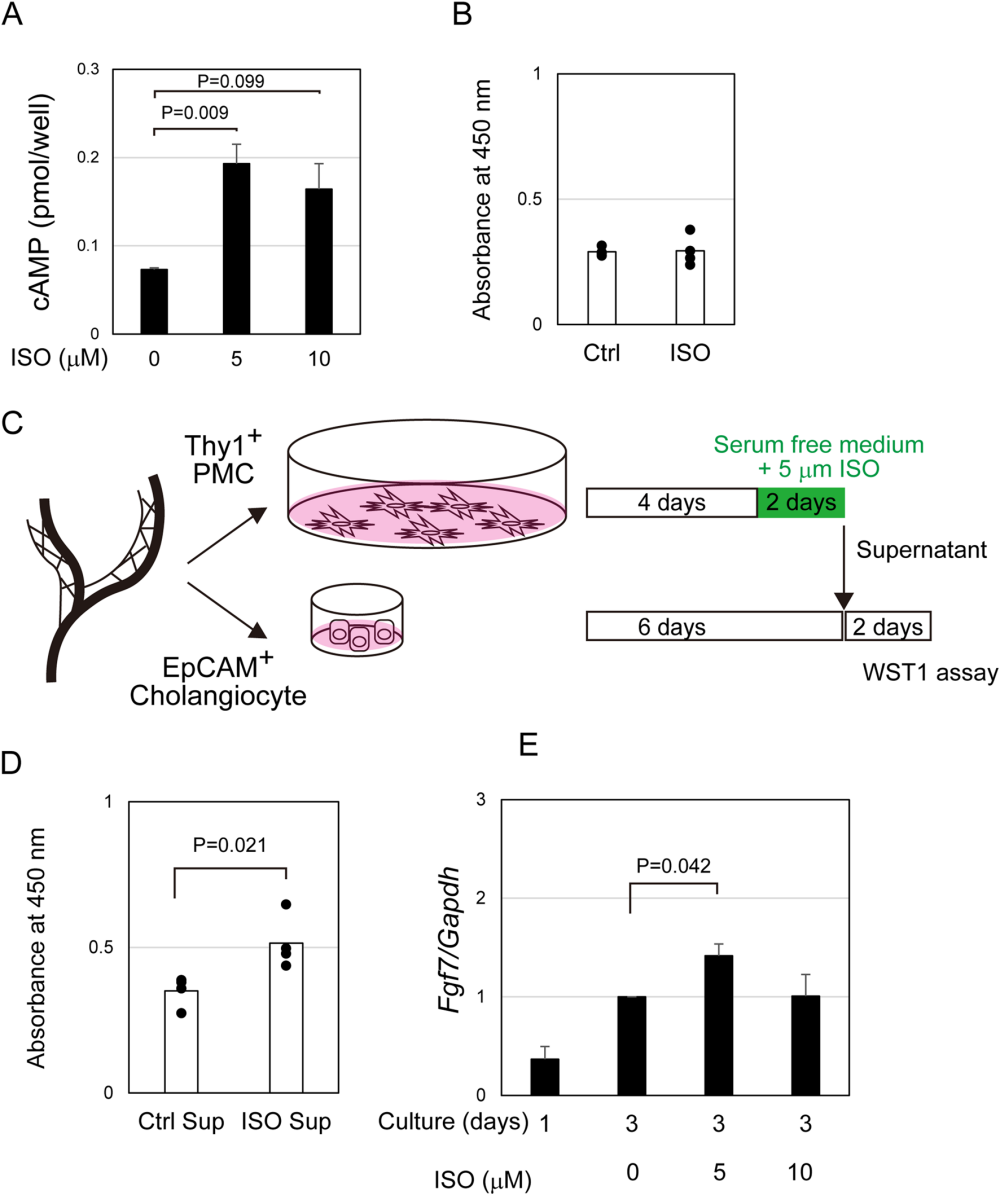
Activation of beta-adrenergic receptors (β-ADRs) induces periportal mesenchymal cells to produce growth factor for cholangiocytes. (**A**) Isoproterenol (ISO) increases intracellular cAMP in PMCs. Intracellular cAMP levels are significantly increased in the presence of 5 μM ISO. The average values of three wells (n = 3) of each condition are shown in the graph. Error bars represent SEM. (**B**) ISO does not affect PMC proliferation. Thy1^+^ cholangiocytes were cultured for a week with or without 10 μM ISO. Cells were further incubated in the presence of WST-1 for 60 min, and then the absorbance at 450 nm was measured. The average value of three wells (n = 3) of each condition are shown in the graph. Bars represent SEM. (**C**) Examination of the effect of Thy1^+^ PMCs culture supernatant on cholangiocyte proliferation. The supernatant of Thy1^+^ PMCs were collected and then added to the culture of EpCAM^+^ cholangiocytes. The proliferation of choalngocytes were examined by WST1 assay. (**D**) PMCs produce soluble factors promoting cholangiocyte proliferation. Cholangiocytes proliferate more efficiently in the culture supernatant of PMCs stimulated by ISO as compared with the control supernatant. (**E**) ISO stimulates the expression of *Fgf7* in PMCs. *Fgf7* expression in PMCs is increased in the presence of 5 μM ISO. Bonferroni test was performed on Microsoft Excel.

The autonomic nerve system consists of sympathetic and parasympathetic nerves. To visualize the connection between extrahepatic and intrahepatic autonomic nerves, we isolated the esophagus between the diaphragm and the stomach with liver (Fig. 1A-1) and stained with sympathetic- and parasympathetic-specific antibodies against TH and vesicular acetylcholine transporter (VAChT), respectively. Previous reports show that hepatic stellate cells (HSCs) in culture produce adrenaline^21^, suggesting that they express TH, a late-limiting enzyme for adrenaline production. However, PCR analyses show that hepatic cells including HSCs do not express *Th* (Fig. S1A). In addition, TH is distinctly detected on tubulin beta3+ (TUBB3+) nerve fibers (Fig. S1B). Therefore, we used TH as a specific marker for intrahepatic sympathetic nerves. Vagus nerve fibers positive for VAChT (VAChT^+^) formed bundles running in parallel with the esophagus (Fig. 1A-2, yellow arrowheads). TH^+^ sympathetic nerve fibers were parallel with these vagus nerve bundles (Fig. 1A-2, white arrowheads) branching toward and contacting the liver, indicating they entered intrahepatic tissue (Figs. 1A-3 and 5, white arrows). In contrast, VAChT^+^ nerve fibers were barely detected in this area (Figs. 1A-4 and 5). Consistently, we found that intrahepatic nerve fibers positive for the pan-neural marker TUBB3, were also positive for TH (Figs. 1B-1 and 2), whereas VAChT^+^ nerve fibers were not detected (Figs. 1B-3 and 4).

### Activation of β-ADRs in vivo enhances DRs

Considering that intrahepatic sympathetic nerves are abundant in periportal tissue including IHBDs, they may have potential roles in IHBD remodeling. Consistently, sympathetic nerve fibers became more abundant around IHBDs in mice fed with DDC-diet (Fig. S2), which is a chronic liver injury animal model associated with ductular expansions that is called ductular reactions (DRs). To reveal the roles of sympathetic nerves in bile duct remodeling during liver injury, ISO, an agonist of β adrenergic receptors (β-ADRs), was used to promote sympathetic stimulation in the liver as the expression of TH, the rate-limiting enzyme for adrenaline synthesis, indicates adrenaline production by intrahepatic nerves. ISO was administered daily to mice by subcutaneous injection for a week, followed by the initiation of DDC-feeding (Fig. 2A). During DDC-feeding, ISO was administered every 5 days. ISO treatment had no effect on liver/body weight ratio and the degree of liver injury (Fig. 2B). After 2 weeks of DDC-feeding, the degree of DRs was examined by qPCR and histochemical analysis. The extent of DRs can be estimated by the increased expression of bile duct markers including osteopontin (*Opn*) and cytokeratin 19 (*Krt19*) (Fig. 2C). We found that ISO administration slightly increased *Opn* expression induced by DDC-diet, whereas it significantly upregulated *Krt19* expression by two-fold. Additionally, we analyzed liver tissue sections stained with anti-CK19 antibody and found that ISO administration increased the ratio of CK19^+^ area per field (Figs. 2D and E) as well as the ratio of Ki67^+^ cells in CK19^+^ ductular structures (Fig. 2F). On the other hand, chemical denervation by administration of 6-hydroxydopamin (6-OHDA) suppressed DRs induced by DDC-diet (Fig. S3). These results suggest that activation of intrahepatic sympathetic nerves accelerates DRs induced by DDC-diet by promoting proliferation of cholangiocytes.

### Sympathetic nerve endings are located near bile ducts

To clarify whether sympathetic nerves directly affect cholangiocytes or not, we stained liver tissue sections with an antibody against synaptophysin (SYP), which is a marker for synaptic vesicles, in combination with a sympathetic nerve marker, TH. Although *Syp* is detected in endothelial cells (ECs), HSCs, PMCs, and cholangiocytes (Fig. S1A), the positivity of SYN on TH^+^ nerve fibers shows the existence of synaptic vesicles. We found that TH^+^/SYP^+^ nerve endings were adjacent to the basal domains of EpCAM^+^ cholangiocytes (Figs. 3A-1 and 2). Additionally, TH^+^SYN^+^ structures were also detected on cells surrounding EpCAM^+^ large duct structures. As large ducts are associated with PMCs positive for Thy1, we stained the liver section with anit-Thy1 antibody, and found that TH^+^SYP^+^ nerve endings were adjacent to Thy1^+^ PMCs (Figs. 3A-3 and 4). To further examine the spatial arrangement between sympathetic nerves and IHBDs, the ratios of cholangiocytes and PMCs which are associated with nerve endings were determined (Fig. 3B). About 30% and 15% of cholangiocytes in small and large IHBDs were associated with SYP^+^ nerve endings. On the other hand, 50% of PMCs surrounding large IHBDs were associated with nerve endings. These results suggest that sympathetic nerves directly regulate bile duct remodeling in peripheral liver tissue where small ductules are abundant, whereas they also indirectly control the remodeling in the hilum region by affecting PMCs as well as cholangiocytes in large ducts (Fig. 3C).

### Activation of β-ADRs induces biliary remodeling in vitro

Immunohistochemical data (Fig. 3) suggest that the activation of β-ADR on cholangiocytes and PMCs is involved in DR promotion induced by ISO. Since ADRs are associated with G-proteins regulating adenylyl cyclase, we tested whether ISO increases intracellular cAMP of cholangiocytes as well as PMCs and then examined possible mechanisms how it affects cholangiocyte proliferation.

First, we measured intracellular cAMP levels in cholangiocytes in the presence of ISO. Primary cholangiocytes were cultured in 96-well plates coated with laminin-111 for 5 days, and then incubated with ISO for an additional 2 days. We found that ISO significantly increased cAMP levels in cholangiocytes (Fig. 4A), providing further support that they directly receive adrenaline secreted from TH^+^ sympathetic nerve endings.

DRs are associated with increased cholangiocyte proliferation. However, when primary cholangiocytes cultured on laminin-coated dish, ISO did not directly promote cholangiocyte proliferation, which was examined by WST-1 assay (Figs. 4B and S4). We also examined how ISO modulates the ductular structures of cholangiocytes using a 3D culture system. EpCAM^+^ cholangiocytes were embedded in type I collagen gel and maintained in the presence of 5 ng/ml epidermal growth factor (EGF) and 100 ng/ml hepatocyte growth factor (HGF). After 7 days of incubation, cholangiocytes form organoids with branching structures (Fig. 4C, arrowheads). ISO was added to the culture on day 7. Over the following 2 days, the branching structures of cholangiocytes slightly increased with or without ISO (Fig. 4D, left panel), indicating that branching was unaffected by ISO. In contrast, the lumen of cholangiocyte organoid further expanded in the presence of ISO (Fig. 4D, right panel). Confocal imaging clearly demonstrated that luminal structures of the organoids were markedly expanded by ISO exposure (Fig. 4E), and the ratio of Ki67^+^-proliferating cholangiocytes was significantly increased in the presence of ISO (Figs. 4E and F). Moreover, lumen expansion induced by ISO proceeded to increase of the cells surrounding the lumen in 3D culture (Fig. S5). Thus, the primary effect of ISO may be increased fluid transport into the luminal space, thereby promoting cholangiocyte proliferation. This conclusion is further supported by the findings that ISO administration during DDC-feeding expanded the luminal space in IHBDs (Fig. S6).

Next, we examined whether ISO directly acts on PMCs. Thy1^+^ PMCs were cultured for 5 days and then incubated in the presence of ISO for an additional 2 days. ISO increased intracellular cAMP levels, indicating that it can directly act on PMCs (Fig. 5A) though it did not affect their proliferation (Fig. 5B). As a possible mechanism for that ISO stimulation on PMCs promoting cholangiocyte proliferation, PMCs may secrete soluble factors acting on cholangiocytes. The supernatant of PMC culture without or with ISO (Control (Ctrl)-Sup and ISO-Sup) was collected and added to the culture of cholangiocytes (Fig. 5C). The result indicated that the supernatant of PMC-ISO-Sup promotes cholangiocyte proliferation more efficiently than that of Ctrl-Sup (Fig. 5D), suggesting that ISO stimulates PMCs to secrete growth factors for cholangiocytes. FGF7 is a candidate of growth factors secreted from PMCs, as it was reported to promote cholangiocyte proliferation in vitro and in vivo (Takase et al., 2013). Consistently, ISO significantly increased *Fgf7* expression in cultured PMCs (Fig. 5E), whereas *Fgf7* expression in the liver of mice fed DDC-diet was only slightly increased (Fig. S7A). FGF7 protein was also increased slightly in PMC-ISO-Sup as compared to the control (Fig. S7B), whereas FGF receptor 2 (FGFR2)-blocking antibody suppressed pro-proliferation effect of PMC-ISO-Sup (Fig. S7C). These results suggest that, in addition to cholangiocytes, ISO stimulates PMCs to express *Fgf7*, which can promote cholangiocyte proliferation.

## Discussion

In this study, we demonstrated that intrahepatic sympathetic nerves regulate DRs induced by chronic liver injury. Our results show that intrahepatic sympathetic nerves act on cholangiocytes and PMCs to expand the apical luminal space and to increase production of growth promoting factors, respectively. We consider that these are underlying mechanisms for DR promotion by administering a β-ADR agonist during DDC-feeding.

Considering the mechanism we proposed in this report, cholangiocyte proliferation may be regulated by a β-ADR agonist or antagonist without liver injury. However, we determined that IHBDs appeared normal and their proliferation was not activated by the administration of ISO in the absence of liver injury (Fig. S8). Therefore, in the context of the interaction between nerves and IHBDs, intrahepatic sympathetic nerves have more crucial roles in chronically injured liver compared with healthy liver.

The intrahepatic autonomic nerve system has been implicated in hepatic metabolism^22–24^, and hepatocyte proliferation^25–27^. Furthermore, sympathetic innervation of bile ducts was also reported previously^28,29^ and histochemical analysis has showed that cholangiocytes express four types of ADRs, namely, α1, α2, β1, and β2^30^. The administration of an α1-ADR agonist increased secretin-induced bicarbonate secretion, which led to choleresis via the inositol triphosphate/Ca^2+^/PKC pathway^30^. In this work, we demonstrate that the activation of β-ADRs with ISO promotes the lumen expansion of cholangiocyte organoids in 3D culture in vitro and during chronic liver injury in vivo, resulting in increased proliferation of cholangiocytes (Figs. 2, 4, S5, and S6). The sequential event of lumen expansion following cell proliferation has been reported previously^31,32^. In addition, ISO also acts on PMCs that produce soluble factors including FGF7 to promote cholangiocyte proliferation. FGF7 derived from PMCs was previously identified as a crucial factor promoting cholangiocyte proliferation during DRs^18^. Thus, we currently consider that intrahepatic sympathetic nerves act on cholangiocytes and PMCs to promote their secretory functions and production of growth promoting factors, respectively, and these direct and indirect effects cooperatively promote cholangiocyte proliferation during chronic liver injury.

Bidirectional nerve–blood vessel interactions have been extensively studied in the context of arterialization of blood vessels. On the one hand, nerve-derived VEGF-A and CXCL12 regulate arterial differentiation and branching morphogenesis, respectively, in developing skin^3^. On the other hand, blood vessel-derived growth factors including NGF^33^, neurotrophin 3^34^, endothelins^35^, artemin^36^, and netrin-1^37^ attract and regulate the formation of the nerve network along arteries. Our current results indicate that intrahepatic sympathetic nerves act on cholangiocytes through adrenaline. We previously demonstrated that bile ducts supply NGF for innervation and re-innervation of liver tissue during postnatal development and regeneration after biliary destruction, respectively^9^. Therefore, bidirectional nerve–biliary interactions are crucial for the formation and remodeling of liver tissue architecture.

In addition to innervation to cholangiocytes and PMCs, intrahepatic nerves are close to αSMA^+^ vascular smooth muscle cells as well as ECs of PVs and hepatic arteries (Figs. S9A and S10). Moreover, quantitative PCR analysis showed that both *Adrb1* and *Adrb2* are expressed in HSCs (Fig. S11), and as previously reported, macrophages and HSCs reacted to aminergic neurotransmitters^38,39^. These results suggest that intrahepatic sympathetic nerves may also regulate their cellular characteristics during liver injury and regeneration, though Desmin^+^ HSCs in the parenchyma are not associated with TH^+^ nerve fibers (Figs. S9B and S10). Therefore, we cannot exclude the possibility that the activation or inactivation of β-ADRs on cells other than cholangiocytes and PMCs indirectly affects DRs. For example, sympathetic signals induce contraction of hepatic arteries^40^, the administration of ISO may change hepatic blood flow, thereby affecting DRs. Moreover, although DDC-exposure primarily affects liver tissue, the methods we used in our study can systemically activate and inactivate sympathetic nerves. However, the present results of in vitro experiments clearly demonstrate that the β-ADR agonist directly acted on cholangiocytes and PMCs, suggesting that those reactions eventually promote cholangiocyte proliferation. Thus, we consider that β-ADRs on cholangiocytes and PMCs are major targets of intrahepatic sympathetic nerves during DRs induced by DDC-insult.

In this study, we demonstrate that intrahepatic sympathetic nerves regulate the remodeling of bile ducts mainly through β-ADRs on cholangiocytes and PMCs during 2 weeks of liver injury. In addition to ductular expansion, ISO treatment slightly increased the deposition of collagen I in the DDC model (Fig. S12). Therefore, strong activation of β-ADR may be a risk factor augmenting fibrosis and blocking β-ADR might be useful to suppress fibrosis progression. However, it is necessary to extend the injury period for inducing liver fibrosis, and, in that case, other types of cells innervated by intrahepatic sympathetic nerves may also have crucial roles in long-term injury inducing hepatic steatosis and fibrosis. Therefore, toward understanding the roles of intrahepatic sympathetic nerves in chronic liver injury, we need to investigate how adrenaline regulates each type of hepatic cells as well as cholangiocytes and PMCs in future works.

## Methods

### Mice

Male C57BL6 mice (8-week-old) weighting 24–26 g were purchased from Sankyo Labo Service Corporation, Inc. (Tokyo, Japan). Mice were maintained for a week before starting experiment in environment with constant light/dark cycle (12 h), ambient temperature (23 ± 2 °C), and humidity (40%). During experiments, they were maintained in the same condition. ISO (Kanto Chemical Co., Inc., Tokyo, Japan) was dissolved in phosphate-buffered saline (PBS) at a concentration of 5 mg/ml and subcutaneously administered to mice (25 mg/kg) daily for a week^41^. A DDC-containing diet was made by mixing 0.1% DDC (Sigma-Aldrich) in MF diet (Oriental Yeast Co. Ltd., Tokyo Japan). To induce chronic liver injury, mice were fed DDC-diet ad libitum for 14 days. During DDC-feeding, ISO was further administered every 5 days. Mice were euthanized by isoflurane for sample collection. All animal experiments were performed under approval from the Animal Experiment Committee of Sapporo Medical University. The animal studies are reported with the recommendations in the ARRIVE guidelines. All the relevant institutional guidelines, along with the ARRIVE guidelines were followed during the study.

### Culture materials

Type I collagen gel was purchased from Koken (Tokyo, Japan). Growth factor reduced Matrigel, laminin-111, EGF, and HGF were purchased from Corning (NY). ISO were purchased from Kanto Chemical Co., Inc. (Tokyo, Japan). NPY was purchased from Cayman Chemical (Ann Arbor, MI). DMEM/F-12 medium (Sigma-Aldrich, St. Louis, MO) supplemented with 10% FBS (MP Biomedicals, Santa Ana, CA), 10 mM nicotinamide (Sigma-Aldrich), 1 × 10^−7^ M dexamethasone (Sigma-Aldrich), and 1 × ITS (Gibco, Grand Island, NY) was used as the basic medium. The growth medium was prepared by adding 5 ng/ml EGF and 5 ng/ml HGF to the basic medium.

### Cholangiocyte isolation

Mice were euthanized by inhalation of isoflurane (Pfizer, New York, NY). Liver was digested by two-step collagenase perfusion followed by collagenase/hyaluronidase digestion as previously reported^42^. EpCAM^+^ cholangiocytes were further purified using a magnetic cell sorter (Bergisch Gladbach, Germany) for 3D culture and a FACSAria II (BD Biosciences) for qPCR.

### cAMP assay

EpCAM^+^ cholangiocytes and Thy1^+^ PMCs were plated onto 96-well plates coated with laminin-111 and type I collagen, respectively, at 5,000 cells per well and cultured for 5 days. Cholangiocytes and PMCs were further kept in the presence of ISO for 2 h and then dissolved in 0.1 N HCl. Intracellular cAMP levels were measured using a Direct cAMP ELISA kit (Enzo Life Sciences, Inc., Farmingdale, NY). After the acetylation procedure, cAMP concentration was measured according to the manufacturer’s protocol. The absorbance at 405 nm was measured using an Infinite 2000 (Tecan, Männedorf, Switzerland). Three wells for each condition were used for measuring cAMP and culture was independently repeated twice.

### WST-1 assay

EpCAM^+^ cholangiocytes and Thy1^+^ PMCs cultured for 5 days in 96-well plates were further maintained in the presence of ISO for an additional 2 days. Alternatively, EpCAM^+^ cholangiocytes cultured for 6 days were further incubated in the supernatant of Thy1^+^ PMCs for 2 days. To collect the supernatant, Thy1^+^ PMCs were cultured in a 12 well plate coated with type I collagen for 4 days in medium contacting 10% FBS and then incubated in the medium containing 1% BSA and ITS with or without 5 μM ISO. After centrifugation at 300× g for 5 min, the supernatant was concentrated using Amicon Ultracel-3 k (Millipore, Burlington, MA). The concentrated supernatant was diluted by fivefold with medium containing 1% BSA, 1 × ITS and 1 × 10^−7^ M Dexamethasone (Dex) before use. WST-1 (Roche, Basel, Switzerland) was diluted tenfold in culture medium and 100 μl of the diluted solution was added to each well. Absorbance at 450 nm was measured at 30 min, 1 h, and 2 h using an 800TS absorbance reader (BioTek, Winooski, VT). Four wells for each type of cell were used for measuring absorbance at 450 nm and culture was repeated three times.

### 3D culture of cholangiocytes

EpCAM^+^ cholangiocytes were cultured in type I collagen gel. Each well of a 24-well plate was coated with 50 μl collagen gel and 10,000 cholangiocytes suspended in 100 μl collagen solution on ice were then plated. After incubation at 37 °C for 30 min, 600 μl of basal medium containing 5 ng/ml EGF and 100 ng/ml HGF was added to each well. Seven days after plating, the medium was replaced with that containing 10 μM ISO. The size of cholangiocyte organoids was assessed before ISO addition and 2 days after ISO addition. Three wells were assessed for each condition and four to five organoids in each well were identified and recorded at culture days 7 and 9 using a Keyence BZ-X700 fluorescence microscope (Osaka, Japan). The luminal areas of those organoids on 2D images were measured using a Keyence Image Analyzer. Each culture was independently repeated four times.

### Immunostaining

Adult mouse liver tissue was fixed in PBS containing 4% paraformaldehyde (PFA) at 4 °C. After embedding in OCT compound, frozen 7-μm-thick sections were prepared on a cryostat (Leica, Wetzlar, Germany). Fixed liver tissue blocks were also used for whole mount immunostaining as previously reported^9^. The 3D culture was fixed in PBS containing 4% PFA at 4 °C for 30 min with gentle shaking. After washing with PBS, samples were permeabilized in PBS containing 1% Triton X-100 at room temperature for 30 min. After blocking in Block Ace (DS Pharma Biomedicals Co. Ltd., Osaka, Japan) containing 0.1% Triton X-100, samples were incubated with primary antibodies, followed by incubation with fluorescent dye-conjugated secondary antibodies. Nuclei were counterstained with Hoechst 33342 (Dojindo Laboratories, Kumamoto, Japan). Primary antibodies used for immunostaining are listed in Table S1. Images were acquired using a BZ-X700 fluorescence microscope (Keyence) and a LSM780 confocal laser scanning microscope (Carl Zeiss, Jena, Germany). To assess cholangiocyte proliferation in organoids, one well for each culture condition was used for immunostaining. Three to five organoids in each well were used to count Ki67^+^ cells.

### qPCR

To analyze gene expression in liver tissue, total RNA was extracted from frozen liver tissue blocks derived from mice fed normal chow (n = 6), DDC-diet (n = 6), DDC diet with ISO (n = 6). To examine the expression of *Adrb1 and Adrb2*, EpCAM^+^ cholangiocytes were isolated using a FACSAria II Flow Cytometer, whereas hepatocytes were obtained after two-step collagenase perfusion of a liver cell suspension, followed by centrifugation at 50 × *g* for 1 min. Cell isolation was repeated four times. Total RNA was used for cDNA synthesis. The primers used for qPCR are listed in Table S2. PCR was performed using an ABI Prism 7500 (ThermoFisher Scientific). Statistical comparisons were performed by unpaired two-tailed Student *t*-test using Microsoft Excel. A *p*-value < 0.05 was considered to indicate a statistically significant difference between the two groups.

### Quantification of DRs

Liver sections derived from mice fed DDC-diet (n = 6), and DDC-diet with ISO (n = 6) were stained with rabbit anti-CK19 and rat anti-Ki67 antibodies, followed by incubation with AlexaFluor633-conjugated anti-rabbit and AlexaFluor555-conjugated anti-rat antibodies. To examine the effect of ISO on DRs, eight different fields were selected from each liver section to quantify the CK19^+^ area in each image using Olympus cellSens Dimension. CK19^+^ areas were quantified using cellSens Dimension. The same sections were used to count Ki67^+^/CK19^+^ cells. Statistical comparisons were performed by unpaired two-tailed Student *t*-test using Microsoft Excel. A *p*-value < 0.05 was considered to indicate a statistically significant difference between the two groups.

## Supplementary Information

Supplementary Information.

## Data Availability

The raw datasets generated during the study are available for research purposes from the corresponding author on reasonable request.
