# Supplementary material for: β-adrenergic receptor agonist promotes ductular expansion during 3,5-diethoxycarbonyl-1,4-dihydrocollidine-induced chronic liver injury
Source: Sci Rep. 2023 May 1;13:7084. doi: 10.1038/s41598-023-33882-w (PMC10151327; doi:10.1038/s41598-023-33882-w)
Supplement: Supplementary file 1 — Supplementary Information. [file 41598_2023_33882_MOESM1_ESM.docx]

**Supplementary Tables and Figures**

**Table S1. Primary Antibodies**

| Antibody | Company | Host animal | Method | Dilution |
| --- | --- | --- | --- | --- |
| CD31 | BD Pharmingen | Rat | IF | 1:500 |
| CD31  (PE-Cy7-conjugated) | BD Pharmingen | Rat | FACS | 1:1000 |
| CD45  (APC-Cy7-conjugated) | BD Pharmingen | Rat | FACS | 1:1000 |
| CK 19 | Tanimizu et al. 2003 | Rabbit | IF | 1:2000 |
| EpCAM | BD Pharmingen | Rat | IF | 1:500 |
| EpCAM  (FITC-conjugated) | Biolegend | Rat | MACS, FACS | 1:1000 |
| HNF4α | Santa Cruz Biotechnology | Goat | IF | 1:600 |
| Ki67 | eBiosciences | Rat | IF | 1:500 |
| Synaptophysin | Abcam | Rabbit | IF | 1:1000 |
| TH | Novusbio | Sheep | IF | 1:1000 |
| VAChT | Synaptic Systems | Rabbit | IF | 1:1000 |

**Table S2. Primers used for PCR**

| Gene name |  | Sequence |
| --- | --- | --- |
| *Adrb1* | Sense | 5′-ACCCGAGTGGAAACTAGGC-3′ |
|  | Antisense | 5′-ACCGGAAAGCCAGGTGATA-3′ |
| *Adrb2* | Sense | 5′-GCATGGAAGGCTTTGTGAAC-3′ |
|  | Antisense | 5′-CTTGGGAGTCAACGCTAAGG-3′ |
| *Gapdh* | Sense | 5′-ACC ACA GTC CAT GCC ATC AC-3′ |
|  | Antisense | 5′-TCC ACC ACC CTG TTG CTG TA-3′ |
| *Fgf7* | Sense | 5’-ACAGAACAAAAGTCAAGGAGC-3’ |
|  | Antisense | 5’-CAGGAACACAGCTGATAGGT-3’ |
| *Krt19* | Sense | 5’-AGATTGAGAGAGAACACGCCTTGC-3’ |
|  | Antisense | 5’-TCAGGCTCTCAATCTGCATCTCCA-3’ |
| *Opn* | Sense | 5’-GGAGGAAACCAGCCAAGG-3’ |
|  | Antisense | 5’-TGCCAGAATCAGTCACTTTCAC-3’ |

**Supplementary Figures**

**
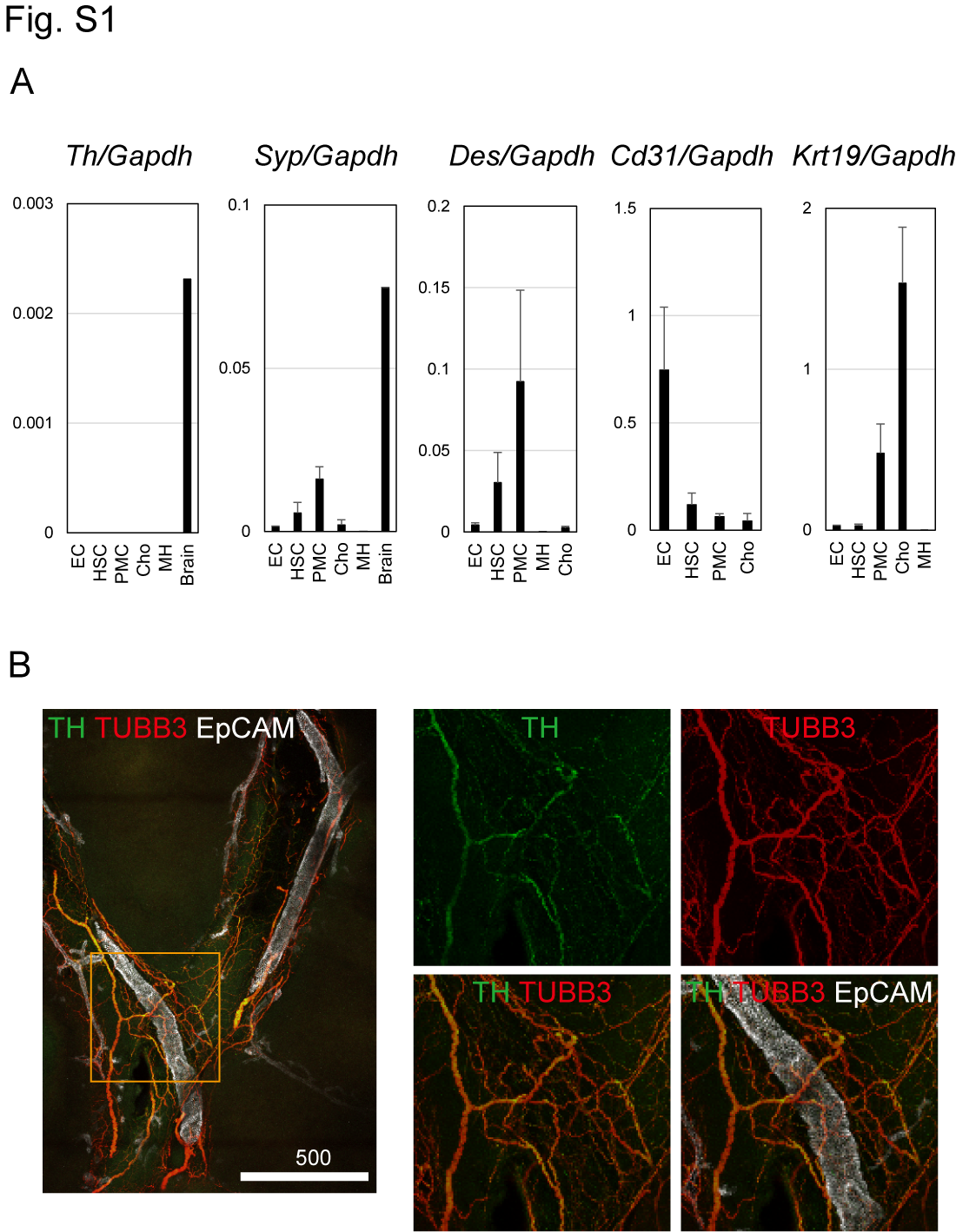
**

**Fig. S1. TH is a marker for intrahepatic sympathetic nerves.**

1. **Tyrosine hydroxylase (*Th*) is not expressed in hepatic cells.** *Th* is not expressed in endothelial cells (ECs), hepatic stellate cells (HSCs), portal mesenchymal cells (PMCs), cholangiocytes (Cho), and mature hepatocytes (MHs). *Synaptophysin* (*Syn*) is weakly expressed in ECs, HSCs, PMCs, and Cho. ECs, HSCs, and Cho were isolated as CD45^-^CD31^+^, CD45^-^Thy1^+^, and EpCAM^+^ cells, respectively, by FACS. MHs were enriched by a low-speed centrifugation (50×*g* for 1min) and HSCs were isolated by density gradient centrifugation. Cells were isolated from healthy adult mice three to five times independently. Expression of *Desmin* (*Des*), *Cd31*, and *Krt19* were examined to confirm the enrichment of HSCs and PMCs, ECs, and Cho, respectively. Brain tissue was used as the positive control for *Th* and *Syn*. Error bars represent SEM.
2. **Tubulin β3^+^ (TUBB3^+^) intrahepatic nerves are positive for TH.** A block of healthy mouse liver was stained with anti-TH, anti-TUBB3, and anti-EpCAM antibodies. After tissue clearing using ScaleViewA2 solution, images were acquired with a confocal microscope.


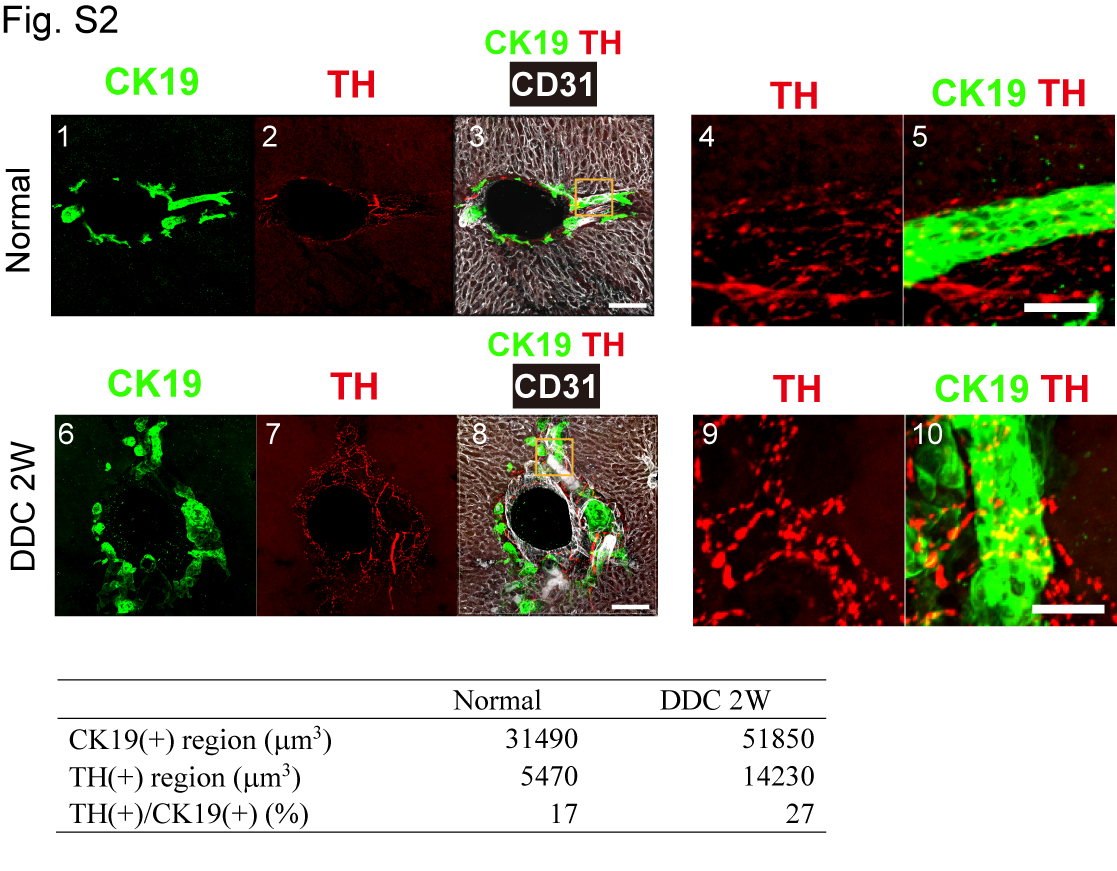


**Fig. S2. DDC-diet induces expansion of ductular structures and sympathetic nerves.**

TH^+^ sympathetic nerves are abundant in the periportal tissue (**panes 1−3**). CK19^+^ bile ducts are closely associated with nerve fibers (**panels 4 and 5**). DDC-treatment induces not only expansion of ductular structures but also nerves fibers (**panels 6−8**). Expanded duct structures are also associated with nerve fibers (**panels 9 and 10**). Boxes in panels 3 and 8 are enlarged in panels (4, 5) and (9, 10), respectively. CK19^+^ and TH^+^ areas in panels (4, 5) and (9, 10) were quantified on Imaris by generating surface models and the results are shown in the table. Scale bars in panels (3, 8) and panels (5, 10) represent 200 and 50 μm, respectively.


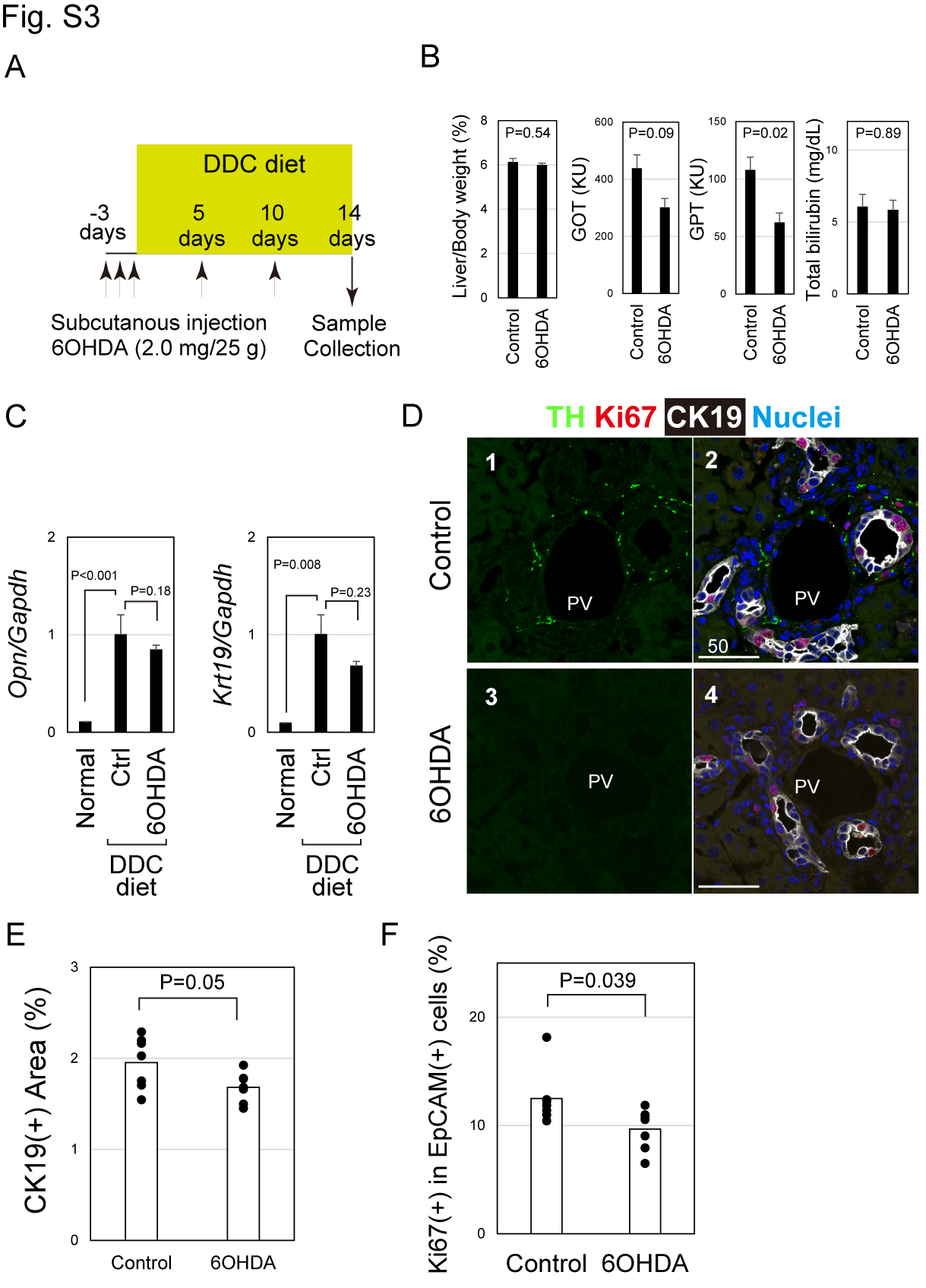


**Fig. S3**. **Pharmacological depletion of sympathetic nerves attenuates ductular reactions (DRs) induced by 3,5-diethoxycarbonyl-1,4-dihydrocollidine (DDC)-mediated injury.**

1. **Timing of chronic liver injury and the administration of 6-hydroxydopamine (6-OHDA).** Mice administered ascorbic acid (n = 7) or 6-OHDA (n = 7) were fed DDC-diet.
2. **6-OHDA administration does not significantly affect hepatic injury induced by DDC-diet.** Liver/body weight is not affected by denervation with 6-OHDA. Serum levels of GOT and total bilirubin were not significantly affected by 6-OHDA administration, whereas serum GPT is slightly decreased. Error bars represent SEM.
3. **Denervation slightly suppresses the increased expression of bile duct cell markers induced by DDC-diet.** DDC-diet increases expression of *Opn* and *Krt19* in liver tissue. The administration of 6-OHDA reduces the expression of *Opn* and *Krt19* but the difference is not statistically significant. Error bars represent SEM.
4. **The expansion of CK19^+^ ducts is suppressed around portal veins (PVs) devoid of sympathetic nerves.** Periportal tissue contains TH^+^ sympathetic nerves in the control (**panels 1 and 2**), whereas periportal tissue lacking sympathetic nerves is observed in mice administered 6-OHDA (**panels 3 and 4**). Scale bars in panels 2 and 4 represent 50 μm.
5. **Quantification of CK19^+^ duct expansion.** The ratio of the CK19^+^ area was determined for each mouse and plotted in the graph. The bars represent the average values of control and 6-OHDA–treated mice.
6. **Denervation with 6-OHDA decreases cholangiocyte proliferation.** Liver sections were stained with anti-TH, anti-CK19, and anti-Ki67 antibodies. The nuclei were counterstained with Hoechst 33342. The ratio of Ki67^+^/CK19^+^ cells to all counted CK19^+^ cells was determined for each mouse and plotted in the graph. The bars represent the average values of control and 6-OHDA–administered mice.


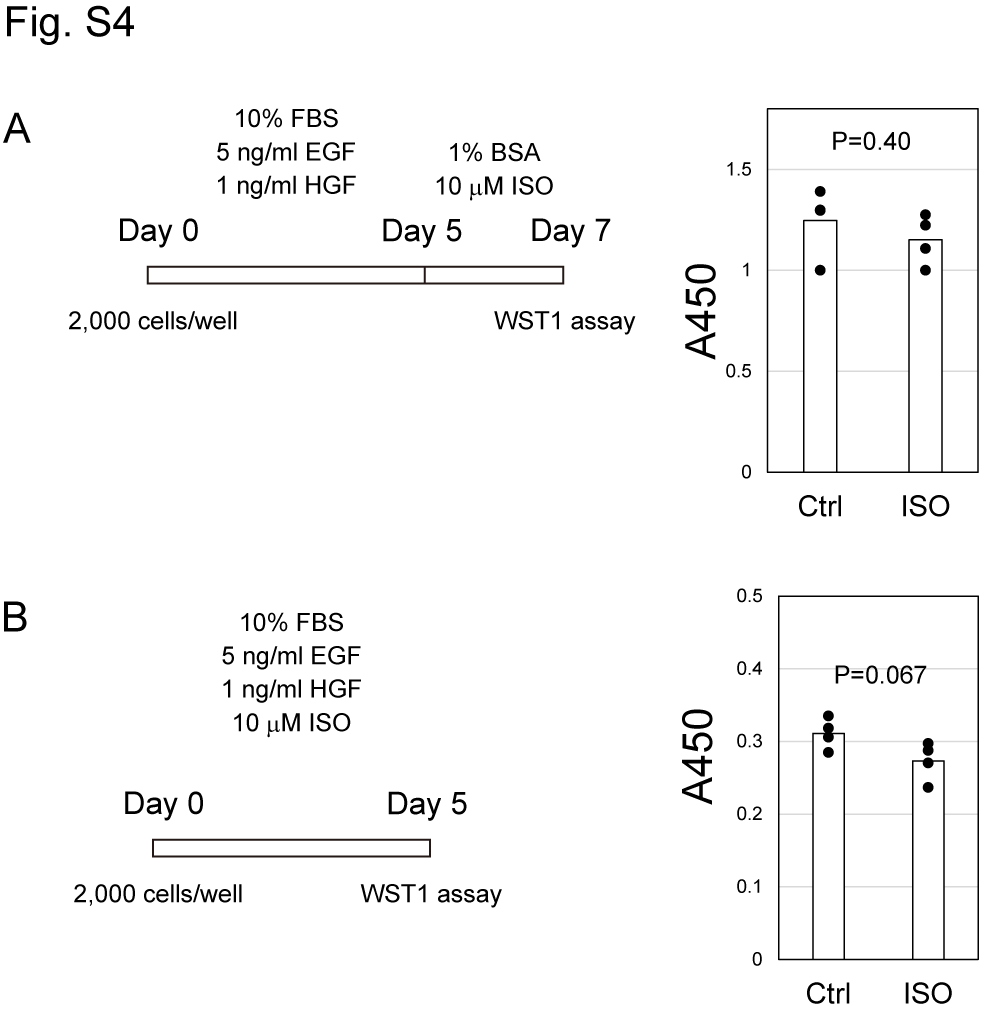


**Fig. S4. ISO does not affect cholangiocyte proliferation.**

ISO was added to the culture of cholangiocytes after 5 days of culture (A) or from the beginning of culture (B). WST1 solution was added to culture and measured absorbance at 450 nm.

**
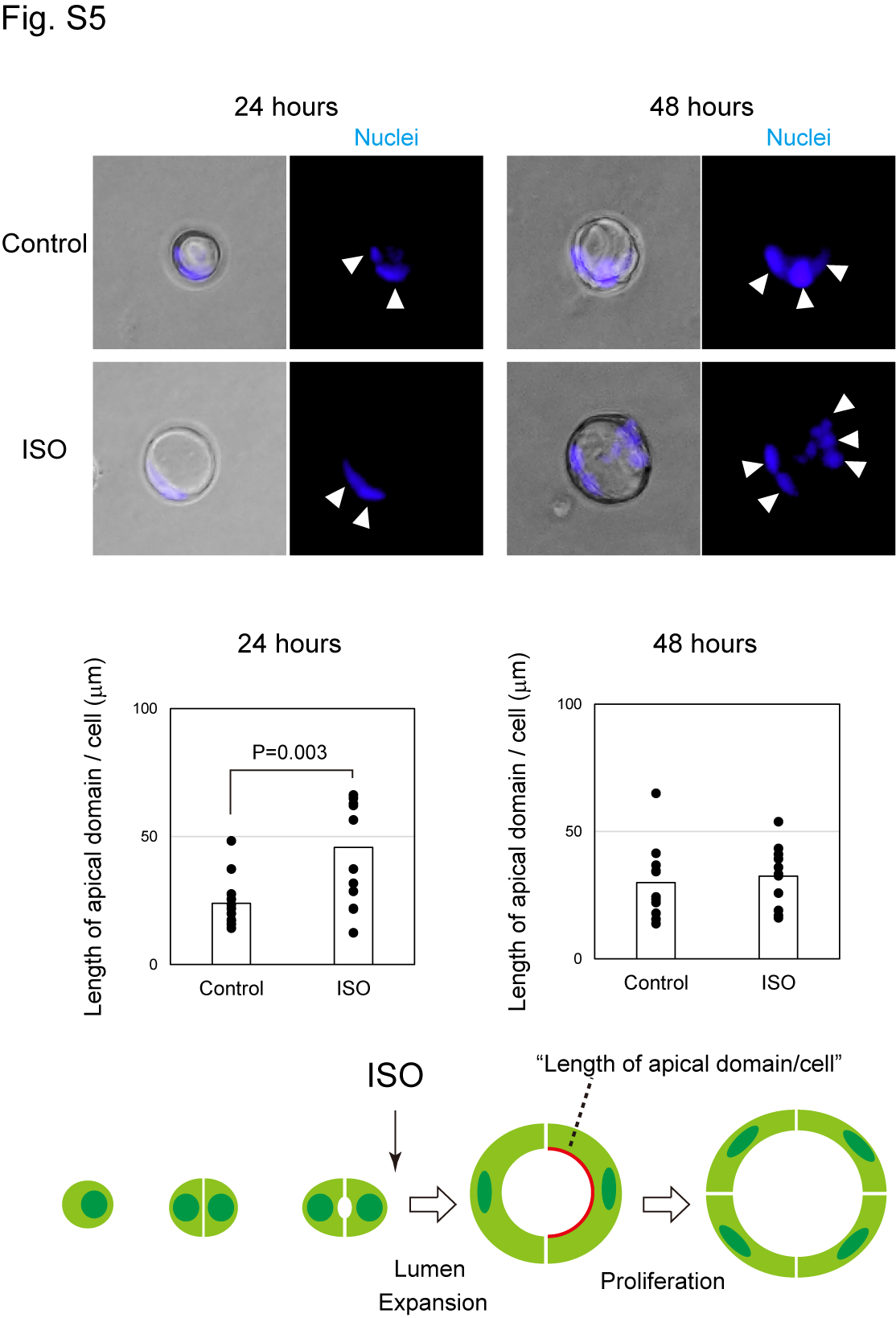
**

**Fig. S5. The luminal expansion of cholangiocyte cysts is followed by proliferation.**

1. **ISO expands the luminal structure of cholangiocyte cysts.** Cholangiocytes form cysts with the central lumen in 3D-culture. ISO promotes lumen expansion during 48 hours. EpCAM^+^ cholangiocytes were isolated from healthy adult mice and expanded on type I collagen gel for a week. They were plated on collagen gel containing 50% Matrigel, and overlaid with collagen gel containing 5% Matrigel. After culture for 5 days, 10 μM ISO was added to the culture and further kept for 24 or 48 hours. Before taking images by a fluorescence microscope, cysts were incubated with 1 μg/ml Hoechst 33342 for 30 mins. Nuclei surrounding the central lumen are indicated by arrowheads.
2. **The lumen expansion precedes cell proliferation.** The length of the apical domain is extended at 24 hours but not at 48 hours after ISO administration. The diameter of lumen was measured and the number of nuclei surrounding the lumen was counted on Olympus cellSens Dimension at 24 and 48 hours after ISO administration. The circumference of the apical lumen was calculated from the diameter of the cyst and divided by the number of cells to indicate the length of the apical domain per cell. Ten cysts were analyzed for the control and the culture with ISO at 24 and 48 hours. Unpaired two-tails *t*-test was performed on Microsoft Excel.

**
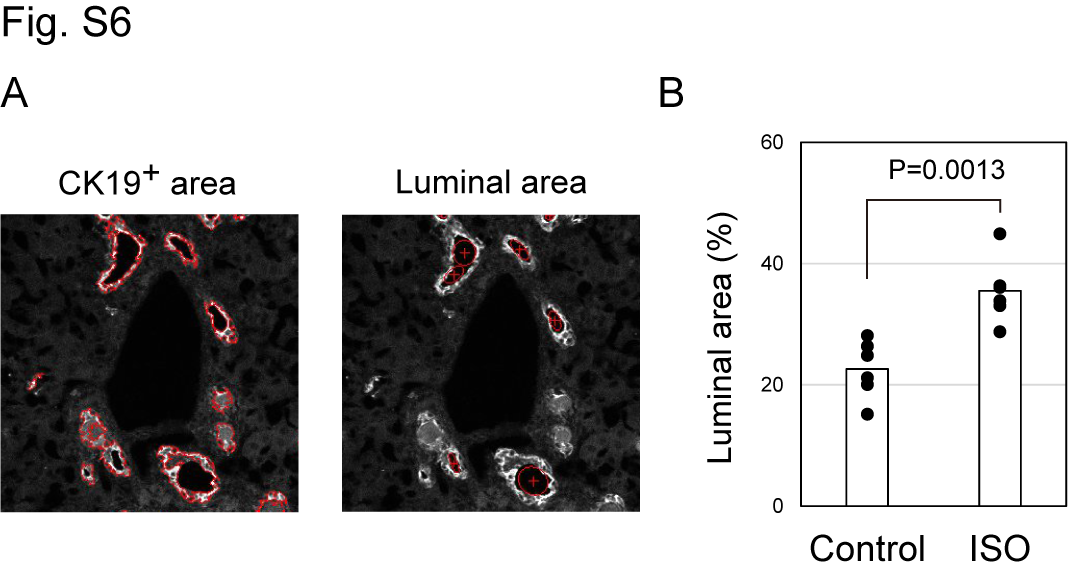
**

**Fig. S6. The luminal size of bile ducts is altered by modulation of sympathetic nerve activation during DDC injury.**

1. **Schematic view for estimation of the luminal size of bile ducts.** The CK19^+^ and luminal areas were quantified on Olympus cellSens Dimension. The luminal area was measured by outlining with ellipse.
2. **The luminal size of CK19^+^ ducts is increased by ISO administration, respectively**. The measurements of CK19^+^ area and the luminal size were performed on four different images. The sum of luminal area in one image was divided by that of CK19^+^ area. Six and seven mice were analyzed to show the effect of ISO and 6-OHDA, respectively. The ratio of the luminal size for each mouse is plotted on the graph. The bars represent the average values of the control and ISO- or 6-OHDA-administrated mice. Unpaired two-tails *t*-test was performed on Microsoft Excel.

**
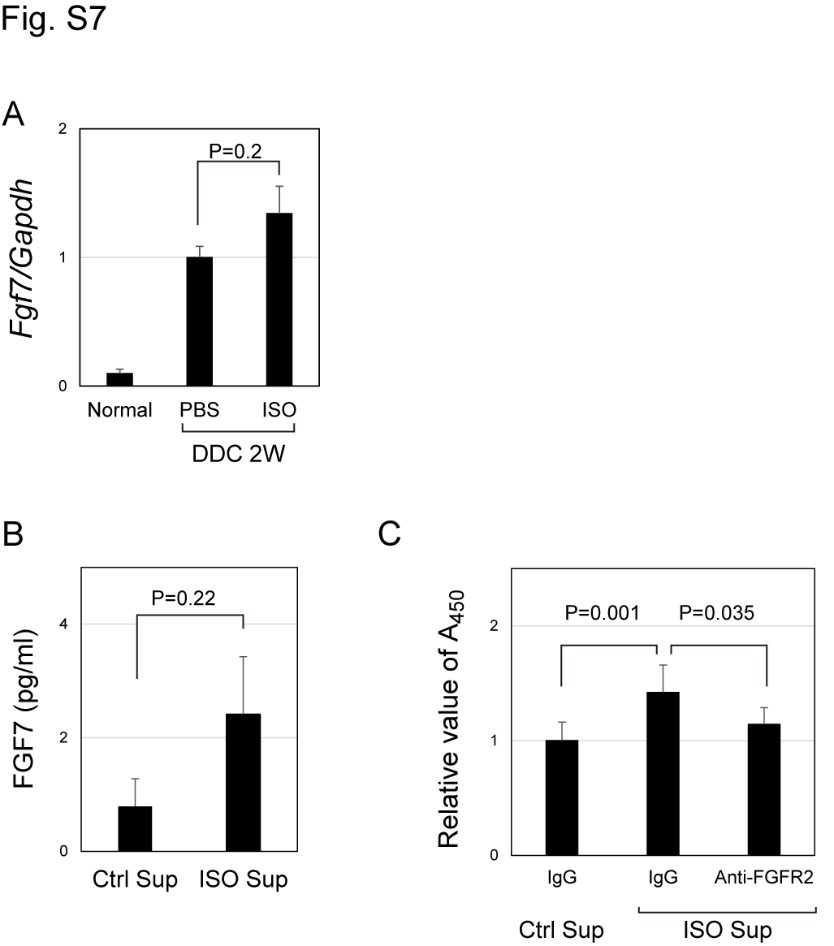
**

**Fig. S7. FGF7 is involved in cholangiocyte proliferation.**

1. **Expression of *Fgf7* in DDC-injured liver administered with ISO.** *Fgf7* is specifically induced by DDC-injury. ISO administration slightly increases *Fgf7* but the difference between the control and ISO-administrated sample is not statistically significant. Bars represent SEM. Unpaired two-tails *t*-test was performed on Microsoft Excel.
2. **FGF7 is secreted to the culture medium of PMCs.** The supernatant was concentrated with an Amicon Ultra 3K by 4-fold and used for ELISA after 10 times dilution. FGF7 is detected in the supernatant with ISO stimulation slightly more compared to the control.
3. **FGFR2-blocking antibody suppresses pro-proliferation effect of PMC ISO-sup.** After 6 days of culture, cholangiocytes were incubated with PMC-sup and 0.1 μg/ml anti-FGFR2 antibody for 48 hours. Bonferroni test was performed on Microsoft Excel.


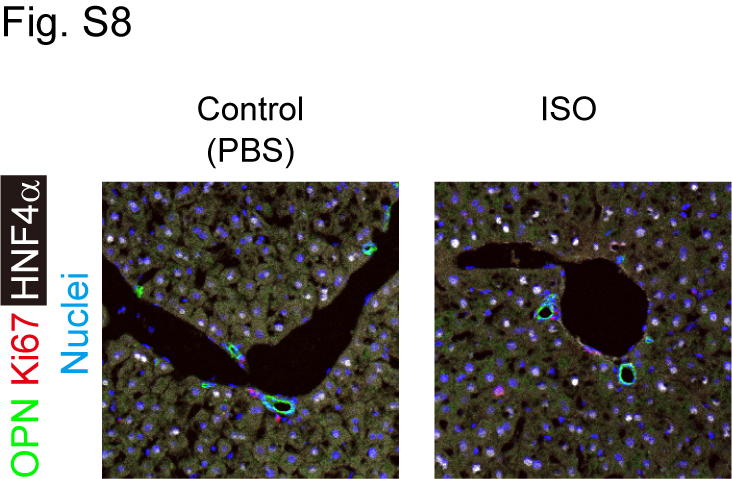


**Fig. S8. Activation of β-ADRs does not affect cholangiocyte proliferation without liver injury.**

Administration of PBS and ISO do not induce expression of Ki67. Mice were subcutaneously administrated with PBS, or ISO according to the schedule same as that during DDC-feeding. Three mice for each condition were analyzed. The representative image of a mouse liver is shown in this figure.


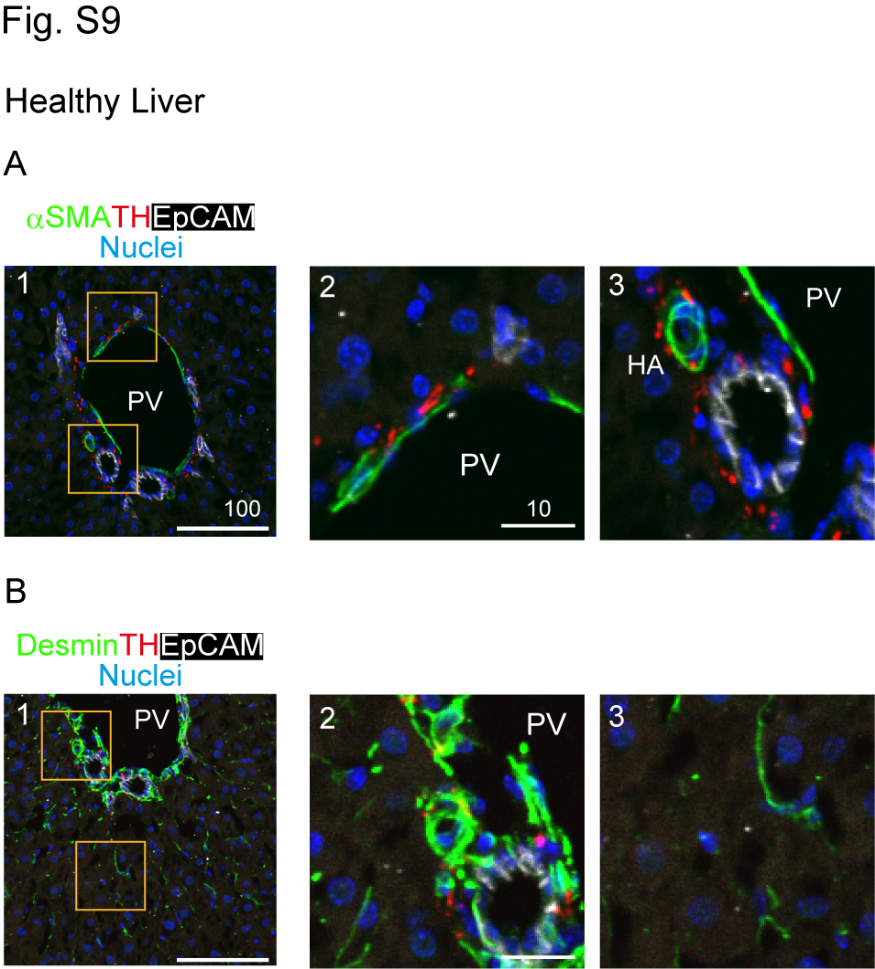


**Fig. S9. Intrahepatic nerves are close to periportal hepatic cells in a healthy liver.**

1. **Intrahepatic nerves are close to vascular smooth muscle cells.** TH^+^ nerves are observed adjacent to αSMA^+^ cells next to PV (panel 2) and hepatic arteries (HA, panel 3) that are considered to be vascular smooth muscle cells. A liver section was stained with Rabbit anti-aSMA (green), sheep anti-TH (red), rat anti-EpCAM (white) antibodies and DAPI (blue). Bars in panels 1 and 2 represent 100 and 10 μm, respectively.
2. **Intrahepatic nerves do not exist near desmin+ HSCs in the parenchyma.** TH^+^ nerves are observed adjacent to Desmin^+^ cells that may include PMCs next to PV (panel 2), whereas they are absent from the parenchyma where Desmin^+^ hepatic stellate cells (HSCs) are located. A liver section was stained with Rabbit anti-Desmin (green), sheep anti-TH (red), rat anti-EpCAM (white) antibodies and DAPI (blue). Bars in panels 1 and 2 represent 100 and 10 μm, respectively.


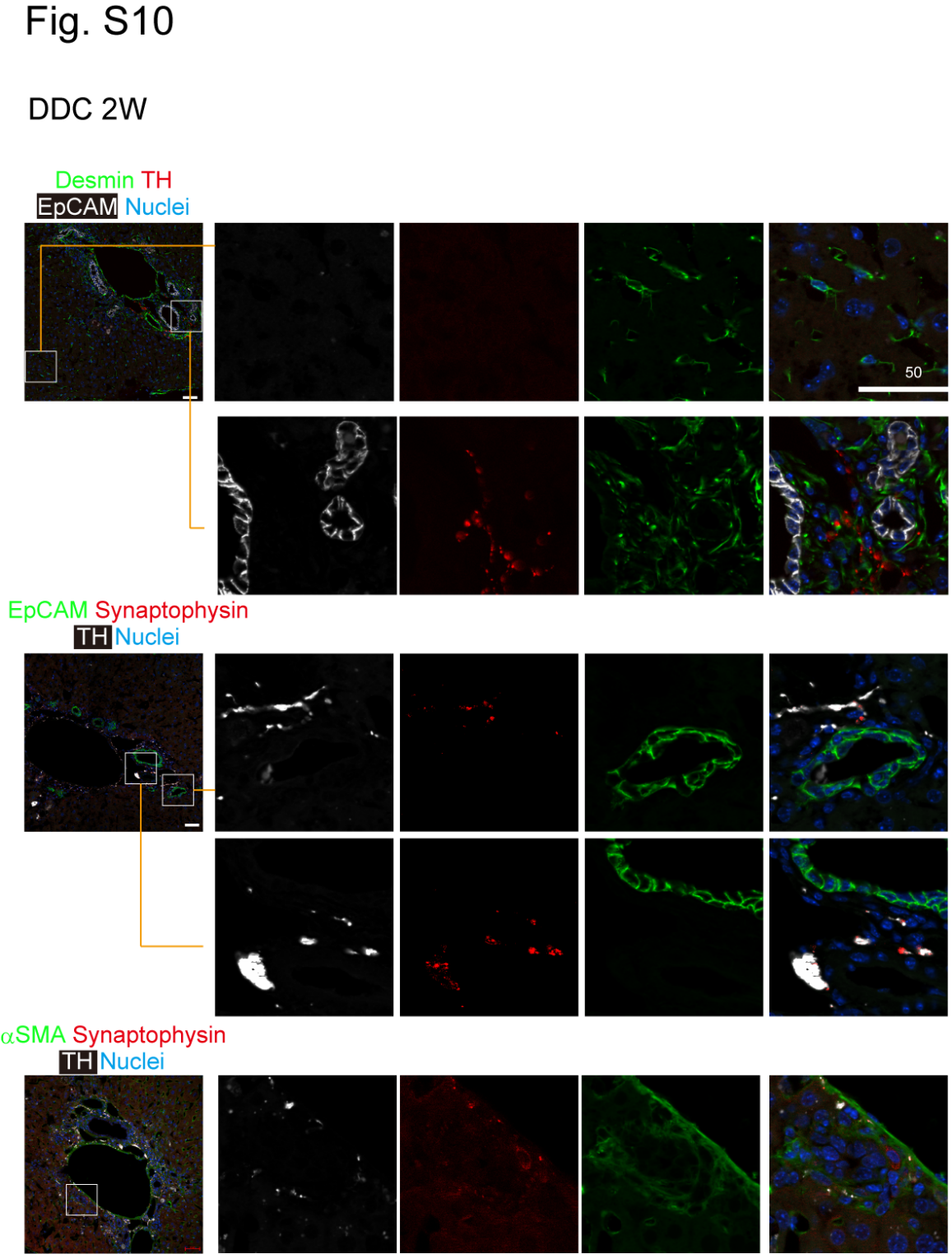


**Fig. S10. Intrahepatic nerves are close to periportal hepatic cells in DDC-injured liver.**

TH^+^ nerves are close to PMCs and cholangiocytes in the periportal areas in the liver of mice fed with DDC-diet for 2 weeks, whereas it does not exist in the parenchymal tissue where HSCs are localized.

**
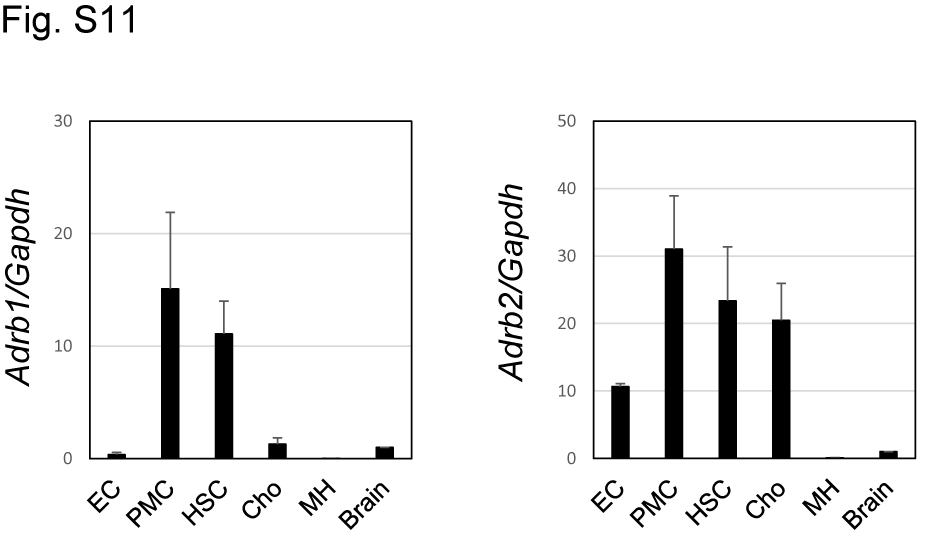
Fig. S11. Expression of β-adrenergic receptors (*Adrb*) in liver cells.**

Cho weakly express *Adrb1*, whereas PMCs and HSCs strongly express it. ECs, PMCs, HSCs, and Cho express *Adrb2*. ECs, HSCs, and cholangiocytes were isolated as CD45^-^CD31^+^, CD45^-^Thy1^+^, and EpCAM^+^ cells, respectively, by FACS. MHs were enriched by a low-speed centrifugation (50×g for 1min) and HSCs were isolated by density gradient centrifugation. Cells were isolated from healthy adult mice three times independently. Error bars represent SEM.


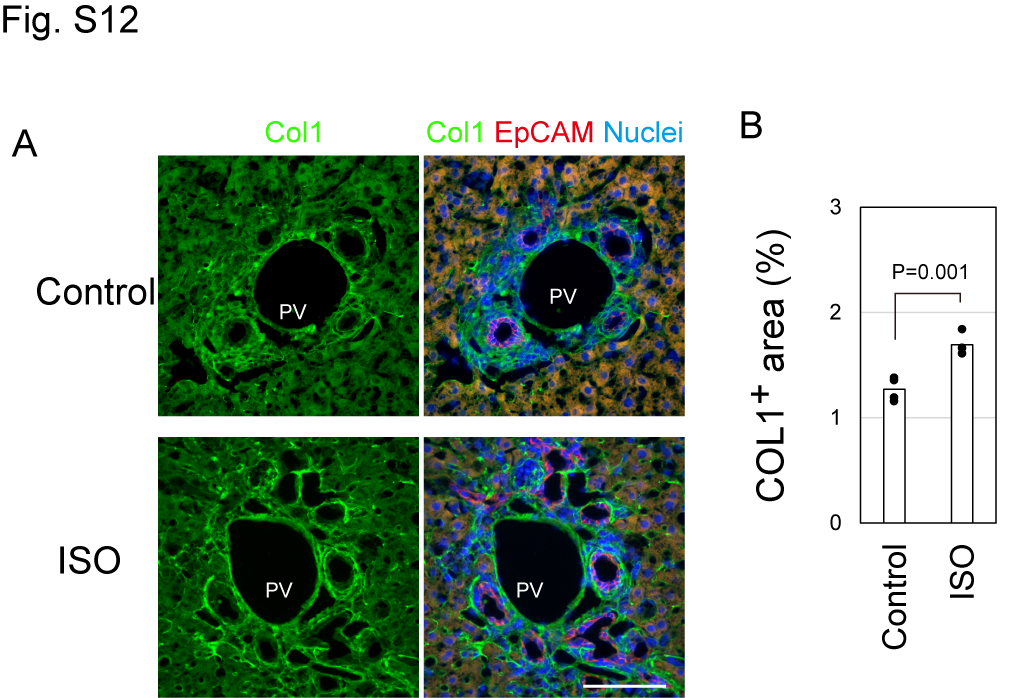


**Fig.S12. Activation of beta-adrenergic receptors (β-ADRs) increases deposition of collagen.**

1. **Deposition of type I collagen (COL1) fibrils is increased by ISO.** COL1 deposition around expanded ductular structures is increased by ISO administration during DDC injury. The scale bar represents 100 μm. PV; portal vein.
2. **COL1^+^ areas are quantitatively increased by ISO administration.** COL1^+^ areas are significantly increased by ISO administration. COL1^+^ area was quantified on ImageJ. Unpaired two-tails *t*-test was performed on Microsoft Excel.
